# Time-Dependent Prognostic Value of the mEASIX Score in Patients with Myelodysplastic Syndromes

**DOI:** 10.3390/diagnostics16111640

**Published:** 2026-05-27

**Authors:** Ahmet Mert Yanık, Sevil Sadri, Ahmet Bahri Şan, Özgür Ömer Gül, Tuba Güllü Koca, Fazıl Çağrı Hunutlu, Yusuf Bilen, Vildan Gürsoy, Vildan Özkocaman, Fahir Özkalemkaş

**Affiliations:** 1 Department of Hematology, Bursa City Hospital, Bursa 16250, Turkey; tubakocamd@gmail.com (T.G.K.); fazilhunutlu@gmail.com (F.Ç.H.); bilenyusuf@hotmail.com (Y.B.); 2 Department of Hematology, Istanbul Training and Research Hospital, Istanbul 34098, Turkey; sevilsadri@hotmail.com; 3 Department of Internal Medicine, Bursa City Hospital, Bursa 16250, Turkey; ahmetbahrisan99@gmail.com (A.B.Ş.); ozguromergul@gmail.com (Ö.Ö.G.); 4 Division of Hematology, Department of Internal Medicine, Faculty of Medicine, Bursa Uludag University, Bursa 16059, Turkey; vildangursoy@uludag.edu.tr (V.G.); vildanoz@uludag.edu.tr (V.Ö.); fahir@uludag.edu.tr (F.Ö.)

**Keywords:** myelodysplastic syndromes, mEASIX, prognosis, overall survival, biomarker, systemic inflammation, endothelial stress

## Abstract

**Background/Objectives:** The modified Endothelial Activation and Stress Index (mEASIX), derived from routinely available laboratory parameters, has emerged as a potential biomarker reflecting systemic inflammatory and endothelial stress. However, its prognostic value in myelodysplastic syndromes (MDS) remains incompletely defined. This study evaluated the association between the mEASIX and overall survival and explored its time-dependent prognostic pattern. **Methods:** This retrospective study included 151 patients with MDS. The mEASIX score was calculated using C-reactive protein, lactate dehydrogenase, and platelet count and was log-transformed for regression analyses. Overall survival was estimated using the Kaplan–Meier method. Univariable and multivariable Cox proportional hazards models were used to evaluate prognostic factors, including age, IPSS-R risk group, hypomethylating agent (HMA) exposure, and venetoclax exposure. The proportional hazards assumption was assessed, and landmark analyses were performed at the median follow-up time (29 months). Receiver operating characteristic analysis was used to determine the optimal mEASIX cut-off value for mortality prediction. Model comparison, internal validation, and sensitivity analyses were also performed. **Results:** During a median follow-up of 29 months, 85 deaths occurred. The mEASIX yielded an AUC of 0.767 for mortality prediction (95% CI 0.691–0.844; *p* < 0.001). Patients with a high mEASIX had inferior overall survival (log-rank *p* < 0.001). In multivariable Cox regression analysis including age, IPSS-R risk group, HMA exposure, and venetoclax exposure, log(mEASIX) remained independently associated with inferior overall survival (HR 1.325, 95% CI 1.166–1.506; *p* < 0.001). In landmark analyses, this association persisted during the early follow-up period (≤29 months; HR 1.347, 95% CI 1.172–1.548; *p* < 0.001) but was attenuated during the late period (>29 months; HR 1.287, 95% CI 0.930–1.782; *p* = 0.128). **Conclusions:** The baseline mEASIX was independently associated with overall survival in patients with MDS and appeared most prognostically relevant during early follow-up. These findings suggest that the mEASIX may complement established risk models, although further validation is needed before broader clinical use.

## 1. Introduction

Myelodysplastic syndromes (MDS) constitute a heterogeneous group of clonal hematopoietic stem cell disorders characterized by ineffective hematopoiesis, peripheral cytopenias, and a variable risk of progression to acute myeloid leukemia (AML), and they predominantly occur in older adults [1,2].

The clinical course of MDS is highly variable, ranging from indolent disease with prolonged survival to rapidly progressive forms associated with significant morbidity and mortality. Therefore, accurate risk stratification is essential for guiding therapeutic decision-making and predicting clinical outcomes [3,4].

Several prognostic scoring systems have been developed for MDS, with the International Prognostic Scoring System (IPSS) and its revised version (IPSS-R) remaining the most widely used in clinical practice [3,5]. Although the IPSS-M improves risk stratification by incorporating molecular data, such testing is not universally available in routine practice, highlighting the need for simple biomarkers based on routine laboratory parameters [6]. These models incorporate bone marrow blast percentage, cytogenetic abnormalities, and peripheral blood cytopenias to assign risk groups but may not fully capture other biological processes that influence disease progression and survival [7,8].

The Endothelial Activation and Stress Index (EASIX) was developed as a scoring system to predict survival in patients undergoing allogeneic hematopoietic stem cell transplantation by reflecting endothelial damage associated with acute graft-versus-host disease [9].

Previous studies have shown that the mEASIX, a modified form of the EASIX in which creatinine is replaced by CRP to better reflect systemic inflammation, predicts ICANS risk after CAR-T cell therapy and has also emerged as a prognostic marker in classical Hodgkin lymphoma [10,11]. Data on the prognostic value of the mEASIX in MDS remain limited, although the original EASIX score has been reported to predict survival in patients with lower-risk disease [12]. This score may be relevant in MDS because systemic inflammation, immune dysregulation, and bone marrow niche alterations contribute to ineffective hematopoiesis and leukemic progression [13,14]. Therefore, this study aimed to evaluate the association of the mEASIX, a simple laboratory-based score, with overall survival and its time-dependent prognostic value in myelodysplastic syndromes.

## 2. Materials and Methods

### 2.1. Study Design and Patient Selection

This retrospective observational study included patients diagnosed with MDS who were followed at our center between 2018 and 2025. Demographic characteristics, clinical data, laboratory parameters, and follow-up information were obtained through a review of electronic medical records.

Patients with complete clinical and laboratory data at the time of diagnosis and with available follow-up information were included in the study. Exclusion criteria comprised an uncertain diagnosis of MDS based on bone marrow examination, the presence of concomitant hematologic disorders or active solid organ malignancies, and irregular follow-up or inaccessible hospital records. Initially, a total of 174 patients were screened for eligibility. After excluding 23 patients due to missing baseline laboratory parameters or insufficient follow-up data, a final cohort of 151 patients was included in the analysis.

Clinical variables collected at the time of diagnosis included age, sex, comorbidities, bone marrow blast percentage, cytogenetic risk classification, specific treatment modalities received (e.g., supportive care, hypomethylating agents, etc.), and MDS subtype. Laboratory parameters included complete blood count, serum lactate dehydrogenase (LDH), creatinine, CRP, ferritin, and other routine biochemical parameters. In addition, transfusion requirements, bone marrow fibrosis grade, and disease transformation to acute myeloid leukemia during follow-up were recorded. Causes of death were obtained from medical records and categorized as AML-related, infection without leukemic transformation, or other causes.

For each patient, prognostic risk assessment was performed using the IPSS and the IPSS-R. The mEASIX score was calculated using routinely available laboratory parameters (mEASIX = lactate dehydrogenase [LDH; U/L] × C-reactive protein [CRP; mg/dL]/platelet count [×10^9^/L]) to investigate its association with survival outcomes in patients with MDS.

During the follow-up period, treatment strategies were guided by IPSS-R risk stratification and individual clinical status. Patients with higher-risk MDS were primarily treated with hypomethylating agents (HMAs). Patients who progressed to AML received either conventional induction chemotherapy or HMA-based regimens combined with a BCL-2 inhibitor (e.g., venetoclax) when intensive chemotherapy was deemed inappropriate. Conversely, patients with lower-risk disease were predominantly managed with supportive care, including transfusion support and symptom-directed treatment. To account for treatment heterogeneity, HMA exposure and venetoclax exposure were included in Cox regression analyses as treatment-related covariates, as these represented the main disease-modifying treatment approaches in this cohort. HMA and venetoclax exposure were defined as receipt of the respective treatment at any time during follow-up. For patients with available data, the time from diagnosis to treatment initiation was calculated descriptively to provide additional context for treatment timing.

### 2.2. Study Outcomes

The primary endpoint of the study was overall survival (OS). OS was defined as the time from the date of diagnosis to death from any cause or last follow-up. Secondary analyses evaluated the association between the mEASIX score and established prognostic factors, including IPSS-R risk categories, laboratory parameters, and clinical characteristics.

### 2.3. Statistical Analysis

All statistical analyses were performed using IBM SPSS Statistics for Windows, Version 25.0 (IBM Corp., Armonk, NY, USA) except for the paired DeLong comparison of ROC curves, which was performed using R software version 4.6.0 (R Foundation for Statistical Computing, Vienna, Austria). All tests were two-sided, and a *p* value < 0.05 was considered statistically significant. Continuous variables were compared using the independent-samples *t*-test or Mann–Whitney U test, depending on their distribution (Shapiro–Wilk test), and were reported as mean ± standard deviation or median (interquartile range). Categorical variables were assessed using Pearson’s chi-square, Fisher’s exact, or linear-by-linear association tests.

The Kaplan–Meier method was used to estimate OS, and survival distributions were compared using the log-rank test. Follow-up duration was calculated using the reverse Kaplan–Meier method. Receiver operating characteristic (ROC) curve analysis was performed to evaluate the ability of mEASIX and its individual components, including CRP, LDH, and platelet count, to predict mortality. The optimal cut-off values were determined using the Youden index, and the area under the curve (AUC) with corresponding 95% confidence intervals (CIs) was calculated for each parameter. AUC was interpreted as a measure of discrimination and not as an indicator of calibration or clinical utility. The AUCs of mEASIX and platelet count were formally compared using the paired DeLong test for correlated ROC curves. Because of its right-skewed distribution, the mEASIX was log-transformed [log(mEASIX)] for regression analyses. Univariable Cox proportional hazards regression was first performed for candidate clinical, laboratory, prognostic, and treatment-related variables, including HMA exposure and venetoclax exposure. Multivariable Cox proportional hazards models were then constructed to evaluate the independent association of log(mEASIX) with overall survival after adjustment for age, IPSS-R risk group, and treatment-related covariates (HMA and venetoclax exposure). The IPSS-R risk group was entered as an ordinal variable.

No statistical evidence of non-proportionality was observed for log(mEASIX). Therefore, exploratory landmark analyses were performed to descriptively assess whether the association between baseline mEASIX and overall survival differed between earlier and later follow-up periods. The 29-month landmark was selected post hoc because it corresponded to the median follow-up duration of the cohort; it was not intended to represent a biologically defined transition point or a validated clinical threshold. For these analyses, multivariable Cox models including treatment exposure variables were repeated separately for the early (≤29 months) and late (>29 months) periods.

In addition, a Venn diagram was generated in R software version 4.6.0 (R Foundation for Statistical Computing, Vienna, Austria) to visualize the overlap among the selected markers.

### 2.4. Ethical Approval

The study protocol was approved by the local institutional ethics committee (Bursa City Hospital, Approval No: 2024-17/1, Date: 16 October 2024) and conducted in accordance with the principles of the Declaration of Helsinki.

## 3. Results

### 3.1. Patient Characteristics

A total of 151 patients with MDS were included in the study, comprising 94 males (62.3%) and 57 females (37.7%). During a median follow-up of 29 months, 85 patients (56.3%) died, while 66 patients (43.7%) were alive at the last follow-up. Transformation to AML occurred in 24 patients (15.9%) during follow-up. Among the deceased patients, infection without leukemic transformation was the most common cause of death (*n* = 61, 71.7%), followed by AML-related causes (*n* = 21, 24.7%), whereas other causes accounted for three deaths (3.5%). No statistically significant association was observed between the analyzed groups and the distribution of causes of death (Fisher’s exact test, *p* = 0.421). Most patients were not candidates for intensive chemotherapy because of age, frailty, and clinical status, and only four patients underwent allogeneic hematopoietic stem cell transplantation during follow-up.

According to MDS subtype classification, 43 patients (28.5%) had MDS-NOS, 41 (27.2%) had MDS with low blasts, 39 (25.8%) had MDS-IB2, 24 (15.9%) had MDS-IB1, and 4 patients (2.6%) had MDS with isolated del(5q). Baseline characteristics of the study cohort are summarized in Table 1.

### 3.2. ROC Analysis of mEASIX

**Figure 1:**
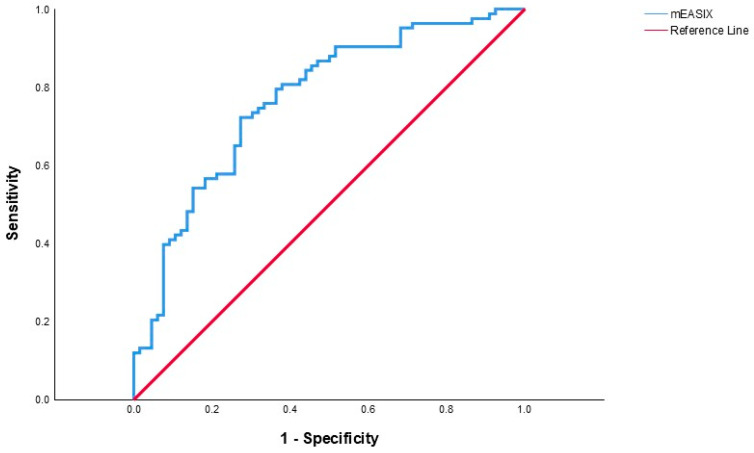
Receiver operating characteristic (ROC) curve of mEASIX for predicting mortality in patients with MDS (AUC: 0.767). ROC, receiver operating characteristic; MDS, myelodysplastic syndromes; mEASIX, modified Endothelial Activation and Stress Index.

ROC analysis was performed to evaluate the mEASIX’s ability to predict mortality. ROC analysis is presented in Figure 1. The AUC was 0.767 (95% CI: 0.691–0.844; *p* < 0.001), indicating good discriminatory performance. An exploratory cut-off value for the mEASIX, derived from the current dataset using the Youden index, was identified as 0.685, corresponding to a sensitivity of 84.7% and specificity of 56.1%. Based on this cut-off value, patients were classified into low-risk (*n* = 50) and high-risk (*n* = 101) groups.

**Figure 2:**
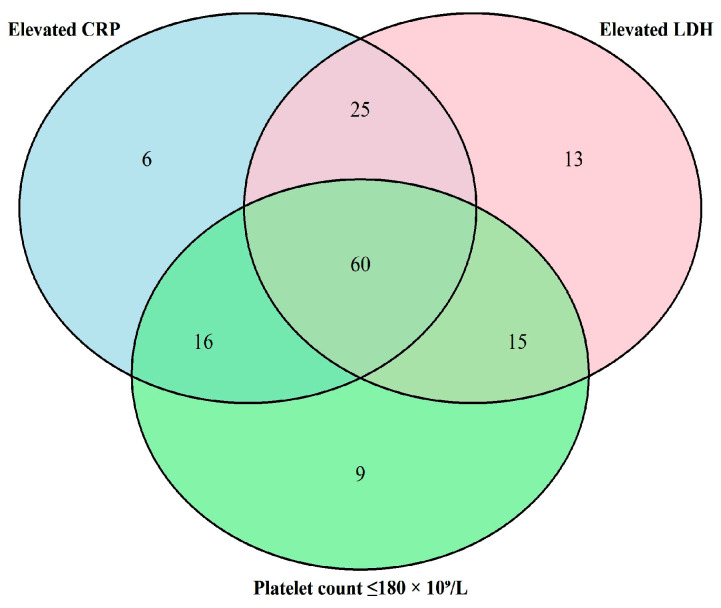
Venn diagram illustrating the distribution and intersection of patients according to the optimal risk thresholds for individual mEASIX components. Thresholds were determined using the Youden index: C-reactive protein > 3.0 mg/dL, LDH > 166.0 IU/L, and platelet count ≤ 180 × 10^9^/L.

To further explore the composite nature of the mEASIX score, we compared its discriminatory performance with that of its individual components. In the paired comparison of correlated ROC curves using DeLong’s test, the AUC of mEASIX was not significantly different from that of platelet count alone (AUC 0.767 vs. 0.730; ΔAUC = 0.037; 95% CI: −0.042 to 0.117; *p* = 0.356). As shown in Table 2, although platelet count, CRP, and LDH were each individually associated with mortality prediction, the composite mEASIX score showed the highest numerical AUC (0.767). However, this was not statistically superior to platelet count alone in the paired DeLong comparison. To descriptively illustrate the overlap among the individual components of mEASIX, patients were categorized using the optimal cut-off values for CRP, LDH, and platelet count. The Venn diagram in Figure 2 showed that elevated CRP (>3.0 mg/dL), elevated LDH (>166.0 IU/L), and low platelet count (≤180 × 10^9^/L) partially overlapped, with 60 patients exhibiting all three abnormalities.

### 3.3. Survival According to mEASIX Risk Groups

Kaplan–Meier analysis showed that patients in the low-risk mEASIX group had significantly longer overall survival than those in the high-risk group (log-rank χ^2^ = 18.017, df = 1, *p* < 0.001). The median overall survival in the high-risk group was 18.4 months (95% CI: 9.8–27.0), whereas it was not reached in the low-risk group.

**Figure 3:**
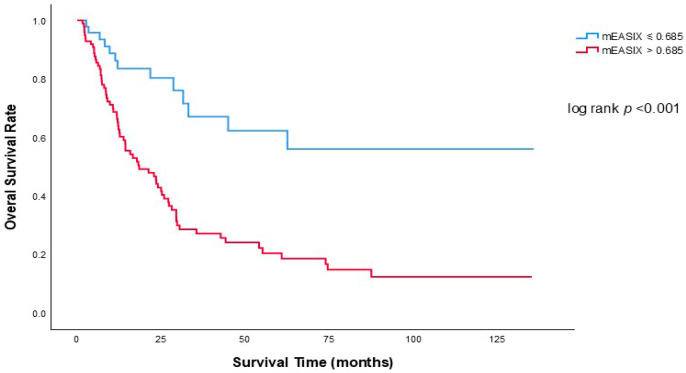
Kaplan–Meier overall survival curves stratified by the mEASIX score (ROC-derived cut-off: 0.685). Median overall survival was 18.4 months (95% CI: 9.8–27.0) in the high-risk group and not reached in the low-risk group (log-rank *p* < 0.001).

In line with this finding, Cox regression analysis showed that the low-risk group had a significantly lower risk of death than the high-risk group (HR = 0.311, 95% CI: 0.172–0.562, *p* < 0.001). Kaplan–Meier survival curves according to mEASIX risk groups are presented in Figure 3.

In an exploratory subgroup analysis restricted to patients in the high and very high IPSS-R risk categories (*n* = 57), patients with high mEASIX scores had numerically inferior overall survival, although this difference did not reach statistical significance (log-rank *p* = 0.088).

### 3.4. Clinical and Laboratory Differences Between mEASIX Risk Groups

As expected from the score components, patients in the high-risk mEASIX group presented with significantly higher LDH levels (*p* = 0.001) and lower platelet counts (*p* < 0.001). Furthermore, the high-risk cohort exhibited significantly elevated bone marrow blast percentages (*p* = 0.017) and higher bone marrow fibrosis grades (*p* = 0.026). Conversely, serum albumin levels were significantly lower in the high-risk group (*p* < 0.001). No significant differences were observed in age at diagnosis; leukocyte, neutrophil, or lymphocyte counts; or in baseline ferritin levels (all *p* > 0.05; Table 3).

### 3.5. Relationship Between the mEASIX and Disease Subtype and Conventional Risk Scores

The distribution of MDS subtypes differed significantly by the mEASIX risk classification (Pearson χ^2^ = 12.177, *p* = 0.016), with MDS-IB1 more frequent in the high-risk group.

Similarly, the distribution of IPSS scores differed significantly between mEASIX groups (Pearson χ^2^ = 12.936, *p* = 0.044). Lower IPSS scores were more common in the mEASIX low-risk group, whereas IPSS scores ≥ 1.0 were more frequently observed in the high-risk group. This association was further supported by a significant linear-by-linear trend (*p* = 0.007).

A significant association was also observed between mEASIX risk groups and IPSS-R categories (χ^2^(4, *n* = 151) = 12.468, *p* = 0.014), indicating concordance between mEASIX-based stratification and established prognostic scoring systems.

### 3.6. Survival According to IPSS-R Risk Categories

**Figure 4:**
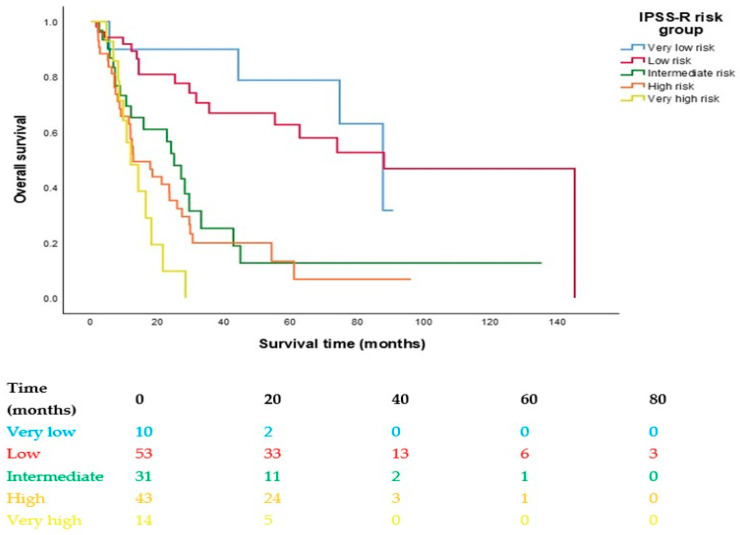
Overall survival according to IPSS-R risk groups. Kaplan–Meier curves showing overall survival stratified by Revised International Prognostic Scoring System (IPSS-R) categories: very low (*n* = 10), low (*n* = 53), intermediate (*n* = 31), high (*n* = 43), and very high risk (*n* = 14). Survival differed significantly across risk groups (log-rank test, χ^2^(4) = 39.774, *p* < 0.001). IPSS-R, Revised International Prognostic Scoring System.

Patients were stratified into five IPSS-R risk categories: very low (*n* = 10), low (*n* = 53), intermediate (*n* = 31), high (*n* = 43), and very high (*n* = 14). Kaplan–Meier analysis demonstrated a progressive reduction in overall survival with increasing IPSS-R risk (log-rank χ^2^ = 39.774, df = 4, *p* < 0.001; Figure 4).

The median overall survival was 87.5 months in the very low-risk group, 87.9 months in the low-risk group, 25.0 months in the intermediate-risk group, 12.7 months in the high-risk group, and 12.0 months in the very high-risk group.

### 3.7. Comparative Prognostic Performance of mEASIX, IPSS, and IPSS-R

Both the IPSS-R and mEASIX scoring systems significantly stratified overall survival in Kaplan–Meier analyses (IPSS-R log-rank χ^2^ = 39.774, *p* < 0.001; mEASIX log-rank χ^2^ = 18.017, *p* < 0.001). Cox regression analyses also showed that increasing risk in both systems was associated with shorter overall survival. In ROC analysis for mortality prediction, the mEASIX yielded an AUC of 0.767 (95% CI: 0.691–0.844; *p* < 0.001), compared with 0.608 (95% CI: 0.519–0.698; *p* = 0.023) for the original IPSS and 0.717 for IPSS-R.

### 3.8. Cox Regression, Time-Dependent Analysis, and Model Validation

In univariable Cox proportional hazards regression analysis, several clinical, laboratory, prognostic, and treatment-related variables were associated with overall survival, including IPSS risk group, bone marrow blast percentage, platelet count, LDH level, albumin level, bone marrow fibrosis grade, HMA exposure, and venetoclax exposure (Table 4).

In the primary multivariable Cox regression analysis including log-transformed mEASIX, age, IPSS-R risk group, HMA exposure, and venetoclax exposure, log(mEASIX) remained independently associated with inferior overall survival (HR 1.325, 95% CI 1.166–1.506; *p* < 0.001). Age (HR 1.040, 95% CI 1.015–1.065; *p* = 0.002), IPSS-R risk group (HR 1.461, 95% CI 1.124–1.898; *p* = 0.005), and HMA exposure (HR 0.423, 95% CI 0.239–0.750; *p* = 0.003) were also independently associated with survival, whereas venetoclax exposure was not statistically significant (HR 1.477, 95% CI 0.811–2.690; *p* = 0.203). Among patients who received treatment (*n* = 79), treatment initiation dates were available for 64 patients (81%). In this subgroup, the median time from diagnosis to treatment initiation was 1.7 months (IQR: 0.9–5.2), indicating that treatment was generally initiated early after diagnosis. The results of the multivariable Cox regression and landmark analyses are summarized in Table 5.

In a separate model comparison analysis including age, IPSS-R category, and log(mEASIX), the addition of log(mEASIX) significantly improved model fit beyond age and IPSS-R alone (likelihood ratio χ^2^ = 19.292, df = 1, *p* = 1.122 × 10^−5^), with an increase in the concordance index from 0.699 to 0.748. This finding was interpreted as evidence of incremental prognostic information rather than established clinical utility. Internal validation of this model using bootstrap resampling (200 repetitions) yielded an optimism-corrected Dxy of 0.4719, corresponding to an optimism-corrected C-index of 0.736. The bootstrap-corrected calibration slope was 0.912.

Given the possibility that the prognostic association of mEASIX might vary over time, landmark analyses were additionally performed at the median follow-up time (29 months). In exploratory landmark analyses using the 29-month landmark, log(mEASIX) remained significantly associated with inferior overall survival during the early follow-up period (≤29 months; HR 1.347, 95% CI 1.172–1.548; *p* < 0.001), whereas this association was attenuated and no longer statistically significant during the later follow-up period (>29 months; HR 1.287, 95% CI 0.930–1.782; *p* = 0.128).

The proportional hazards assumption was assessed using Schoenfeld residual-based tests. No statistical evidence of non-proportionality was observed for log(mEASIX). Therefore, landmark analyses were performed as exploratory descriptive analyses to assess whether the association between baseline mEASIX and overall survival differed between earlier and later follow-up periods. In the separate model including age, IPSS-R category, and log(mEASIX), no evidence of non-proportionality was observed for log(mEASIX) or age, whereas some evidence of non-proportionality was observed for IPSS-R categories, resulting in a significant global test (*p* = 0.036). In additional treatment-adjusted and stratified sensitivity analyses, log(mEASIX) remained significantly associated with inferior overall survival, supporting the robustness of the main findings. Additional model performance, internal validation, and proportional hazards assessment results are summarized in Table 6.

## 4. Discussion

In this retrospective cohort of 151 patients with MDS, the baseline mEASIX was independently associated with overall survival. The association remained significant after accounting for age and IPSS-R risk group. It was also broadly consistent in additional treatment-adjusted and stratified sensitivity analyses, suggesting that the observed prognostic association was not solely attributable to model specification, although residual treatment-related bias cannot be excluded. In landmark analyses, the association was most evident during early follow-up, whereas it was attenuated and no longer statistically significant in the late period.

The EASIX score, initially described as a marker of endothelial stress and a strong predictor of graft-versus-host disease and survival following allogeneic hematopoietic stem cell transplantation, was later modified by substituting CRP, a marker of systemic inflammation, for creatinine [9,10]. This modification resulted in the mEASIX score, which has been evaluated across several disease settings to predict survival and identify high-risk patients who may benefit from intensified clinical management.

The mEASIX score has been shown to be predictive of survival in patients with Hodgkin lymphoma and in pediatric patients with acute leukemia undergoing haploidentical allogeneic hematopoietic stem cell transplantation, and to predict immune effector cell–associated neurotoxicity syndrome (ICANS) in patients with B-cell acute lymphoblastic leukemia (B-ALL) and large B-cell lymphoma (LBCL) receiving CAR-T cell therapy [10,11,15].

Increasing evidence indicates that a subset of patients classified as lower-risk MDS by conventional prognostic scoring systems may in fact carry high-risk molecular features, which may partly explain their unexpectedly poor clinical outcomes [16,17,18]. To our knowledge, limited data exist evaluating the mEASIX score in MDS. The original EASIX, initially developed to assess endothelial vulnerability, was later modified into mEASIX by replacing creatinine with CRP to better reflect systemic inflammatory stress [10]. Previously, Merz et al. demonstrated that classic EASIX predicted survival only in lower-risk MDS, suggesting that it captures cardiovascular complications rather than the leukemia-related mortality that predominates in higher-risk disease [12]. In contrast, our exploratory analysis showed a persistent association between elevated mEASIX and inferior survival in high-risk patients, although statistical significance was not reached (*p* = 0.088), possibly because of limited sample size.

The association between higher mEASIX scores and inferior outcomes may reflect a broader state of systemic inflammatory stress and reduced physiological reserve, as mEASIX integrates CRP, LDH, and platelet count. Systemic inflammation, immune dysregulation, and bone marrow microenvironmental alterations are biologically plausible contributors to adverse outcomes in MDS [13,19]. Iron overload and transfusion-related burden in MDS have traditionally been discussed in relation to cardiovascular morbidity and mortality, providing an important clinical context for systemic vulnerability [20,21,22]. In our cohort, however, deaths were more commonly infection-related, suggesting that the prognostic signal captured by the mEASIX may extend beyond a single complication pathway and may reflect broader baseline vulnerability. This interpretation remains hypothetical, as the present clinical dataset cannot establish the underlying causal pathways, and endothelial biomarkers, cytokine profiling, and mechanistic validation were not available. In this context, the descriptive intersection analysis (Figure 2) showed that elevated CRP, elevated LDH, and low platelet count only partially overlapped across patients, suggesting that these parameters may reflect related but non-identical biological information. Therefore, mEASIX should be interpreted as an exploratory composite marker that may provide complementary prognostic context by integrating inflammatory activity, cellular stress, and hematopoietic reserve. Larger independent cohorts are needed to determine whether this numerical difference translates into clinically meaningful incremental discrimination.

A key finding of our study was the time-dependent prognostic pattern of mEASIX. Landmark analyses showed that the baseline mEASIX remained significantly associated with inferior overall survival during the early follow-up period (≤29 months), even after accounting for age, IPSS-R risk group, HMA exposure, and venetoclax exposure, whereas this association was attenuated in the late period. This temporal pattern suggests that the prognostic relevance of mEASIX may be greatest earlier in the disease course. The association remained evident in treatment-adjusted models, indicating that the early prognostic signal was not solely dependent on unadjusted survival comparisons. However, because treatment exposure was not modeled as a time-dependent covariate and the late-period model was based on a smaller post-landmark cohort, these findings should be interpreted in the context of these methodological considerations. Separate model comparison analyses also showed that inclusion of log(mEASIX) significantly improved model fit beyond age and IPSS-R alone, with better apparent discrimination and acceptable optimism-corrected discrimination after bootstrap validation. These findings suggest a potential complementary prognostic role for mEASIX; however, external validation and dedicated clinical utility analyses are needed before routine clinical use.

Our study also has several strengths. The cohort included a substantial number of events, allowing multivariable survival modeling. Model assumptions were carefully assessed using Schoenfeld residual-based testing, and additional stratified Cox models were used to address the non-proportionality observed mainly for IPSS-R categories. In addition, landmark analysis also provided a clinically interpretable framework for exploring possible temporal variation in the prognostic association of the mEASIX.

However, several limitations should be acknowledged. The retrospective design introduces the possibility of selection bias, and the study’s single-center design limits generalizability. External validation in independent cohorts is therefore required. In addition, molecular data necessary for IPSS-M classification were unavailable for most patients. Treatment heterogeneity was addressed by including HMA and venetoclax exposure as treatment-related covariates in the multivariable models. Although this approach helped account for major treatment-related differences, exposure was captured as a binary receipt during follow-up rather than modeled as a time-dependent covariate. Therefore, we cannot entirely exclude the potential for residual immortal time bias or confounding by indication. Accordingly, these treatment-adjusted analyses are best viewed as exploratory insights, serving to generate hypotheses for future prospective validation. The exploratory landmark analyses provided an additional descriptive perspective on the temporal pattern of the mEASIX–survival association. The 29-month landmark corresponded to the median follow-up duration and allowed comparison of earlier and later follow-up periods; however, the late-period findings should be interpreted as exploratory because fewer patients and events remained at risk. We also acknowledge that the exploratory cut-off of 0.685 was derived from the same cohort used for the survival analyses, which may have introduced optimism bias and increased the risk of overfitting. Therefore, this threshold should be treated as exploratory and not interpreted as a fixed clinical decision boundary. Validation in independent cohorts is required to assess its reproducibility and potential clinical utility in MDS.

Another limitation is that the components of the mEASIX are dynamic and may be affected by conditions other than MDS. CRP and LDH may be transiently elevated by intercurrent inflammation or subclinical infection, whereas platelet count may also be influenced by peripheral consumption, splenomegaly, or treatment-related factors. Because the mEASIX was assessed only at baseline, it may not fully reflect temporal changes in inflammatory and endothelial stress. In addition, inter-institutional variability in laboratory assays may affect reproducibility.

In conclusion, the baseline mEASIX was independently associated with overall survival in patients with MDS and appeared to have its strongest prognostic association during early follow-up. As a biomarker derived from routine laboratory parameters, it may reflect aspects of host-related systemic vulnerability not fully captured by disease-based risk models alone. However, given the retrospective single-center design, treatment heterogeneity, and the exploratory nature of some secondary analyses, these findings should be interpreted cautiously. Further validation in larger, preferably prospective multicenter cohorts is needed before the mEASIX can be more confidently integrated into routine risk assessment. Importantly, the principal prognostic findings of the present study were supported by continuous log(mEASIX) analyses in multivariable and landmark Cox models, rather than relying solely on dichotomization based on the ROC-derived threshold. These findings were also supported by additional treatment-adjusted, stratified, and internally validated model analyses.

## Figures and Tables

**Table 1:** Baseline clinical, hematologic, and prognostic characteristics of the study cohort (*n* = 151).

Variable	Value
Demographics	
Total patients	151 (100%)
Sex	
Male	94 (62.3%)
Female	57 (37.7%)
Disease classification	
MDS with low blasts (MDS-LB)	41 (27.2%)
MDS with increased blasts-1 (MDS-IB1)	24 (15.9%)
MDS with increased blasts-2 (MDS-IB2)	39 (25.8%)
MDS-5q (isolated del(5q))	4 (2.6%)
MDS, not otherwise specified (MDS-NOS)	43 (28.5%)
IPSS risk distribution	
Low risk (Score 1)	115 (76.2%)
Intermediate risk (Score 2)	30 (19.9%)
High risk (Score 3)	6 (4.0%)
IPSS-R risk distribution	
Very low (Score 0)	10 (6.6%)
Low (Score 1)	53 (35.1%)
Intermediate (Score 2)	31 (20.5%)
High (Score 3)	43 (28.5%)
Very high (Score 4)	14 (9.3%)
Comorbidities	
Hypertension	76 (50.3%)
Diabetes mellitus	48 (31.8%)
Coronary artery disease	44 (29.1%)
Clinical outcomes	
Alive at last follow-up	66 (43.7%)
Deceased	85 (56.3%)
AML transformation	24 (15.9%)
Cause of death (*n* = 85)	
Infection without leukemic transformation, *n* (%)	61 (71.7%)
AML-related causes, *n* (%)	21 (24.7%)
Other causes, *n* (%)	3 (3.5%)
Laboratory parameters	
Leukocytes (/µL), median (min–max)	3850 (200–72,470)
Hemoglobin (g/dL), mean ± SD (min–max)	8.48 ± 1.78 (4.30–12.70)
Platelets (/µL), median (min–max)	104,300 (4000–638,000)
LDH (IU/L), median (min–max)	219 (35–756)
Ferritin (ng/mL), median (min–max)	539 (6–7470)

Disease classification was based on the WHO 2022 criteria for myelodysplastic neoplasms. IPSS and IPSS-R categories correspond to the original prognostic scoring systems. Normally distributed variables are presented as mean ± standard deviation, whereas non-normally distributed variables are expressed as median (range). Causes of death were evaluated among deceased patients only (*n* = 85); “other causes” correspond to cardiovascular mortality.

**Table 2:** Diagnostic performance of the mEASIX score and its individual components for predicting overall mortality.

Marker	Optimal Cut-Off	AUC (95% CI)	Sensitivity (%)	Specificity (%)	*p*-Value
mEASIX	>0.685	0.767 (0.691–0.844)	84.7	56.1	<0.001
Platelets	≤180 × 10^9^/L	0.730 (0.647–0.812)	84.7	57.6	<0.001
CRP	>3.01 mg/dL	0.669 (0.580–0.757)	80.0	40.9	<0.001
LDH	>166.0 IU/L	0.638 (0.551–0.726)	78.8	30.3	0.004

AUC, area under the curve; CI, confidence interval; CRP, C-reactive protein; LDH, lactate dehydrogenase; mEASIX, modified Endothelial Activation and Stress Index. Thresholds for individual components were determined using the Youden index.

**Table 3:** Comparison of clinical variables according to mEASIX risk groups.

Parameter	Low-Risk mEASIX (Mean Rank)	High-Risk mEASIX (Mean Rank)	*p*-Value
Age at diagnosis	76.19	75.91	0.970
Bone marrow blasts (%)	63.65	82.11	**0.017**
Leukocyte count	75.66	76.17	0.946
Neutrophil count	75.21	76.39	0.876
Lymphocyte count	77.60	75.21	0.752
Albumin	94.23	66.98	**<0.001**
LDH (IU/L)	58.46	84.68	**0.001**
Bone marrow fibrosis grade	65.07	81.41	**0.026**
Ferritin at diagnosis	69.25	79.34	0.182

Values are presented as mean ranks. Comparisons were performed using the Mann–Whitney U test. LDH and platelet count are components of the mEASIX formula and therefore differ between groups by definition. Bold values indicate statistical significance (*p* < 0.05). LDH, lactate dehydrogenase.

**Table 4:** Univariate Cox regression analysis for overall survival.

Variable	HR (95% CI)	*p*-Value
Coronary artery disease	1.417 (0.89–2.26)	0.142
Hypertension	0.761 (0.50–1.17)	0.212
Diabetes mellitus	1.266 (0.81–1.98)	0.302
COPD	2.899 (0.91–9.26)	0.072
IPSS—Intermediate vs. Low	2.317 (1.40–3.83)	**0.001**
IPSS—High vs. Low	5.299 (2.23–12.62)	**<0.001**
Bone marrow blast (% increase)	1.106 (1.067–1.147)	**<0.001**
Leukocyte count (per 5 × 10^9^/L increase)	1.108 (1.009–1.216)	**0.031**
Neutrophil count (per 10^9^/L increase)	1.023 (0.999–1.047)	0.065
Lymphocyte count (per 10^9^/L increase)	1.107 (0.915–1.339)	0.295
Hemoglobin (g/dL increase)	0.901 (0.80–1.02)	0.088
Hematocrit (% increase)	0.972 (0.94–1.01)	0.156
Platelet count (per 50 × 10^9^/L increase)	0.740 (0.652–0.841)	**<0.001**
Total protein (g/dL increase)	0.968 (0.94–0.997)	**0.028**
Albumin (g/dL increase)	0.921 (0.89–0.95)	**<0.001**
LDH (IU/L increase)	1.003 (1.001–1.004)	**0.005**
Bone marrow fibrosis (grade increase)	1.436 (1.17–1.76)	**0.001**
IPSS-R—Intermediate vs. Very low/Low	3.958 (1.33–11.77)	**0.013**
IPSS-R—High vs. Very low/Low	5.211 (1.81–15.03)	**0.002**
IPSS-R—Very high vs. Very low/Low	8.720 (2.69–28.33)	**<0.001**
HMA exposure	0.248 (0.152–0.404)	**<0.001**
Venetoclax exposure	1.880 (1.070–3.302)	**0.028**

Values in bold indicate statistical significance (*p* < 0.05). Leukocyte, neutrophil, lymphocyte, and platelet counts were rescaled to clinically interpretable units before Cox regression analysis. HR, hazard ratio; CI, confidence interval; COPD, chronic obstructive pulmonary disease; IPSS, International Prognostic Scoring System; IPSS-R, Revised International Prognostic Scoring System; LDH, lactate dehydrogenase; HMA, hypomethylating agent.

**Table 5:** Multivariate Cox regression analysis for overall survival.

Variable	Entire Cohort HR (95% CI)	*p*-Value	Early Period ≤ 29 Months HR (95% CI)	*p*-Value	Late Period > 29 Months HR (95% CI)	*p*-Value
log(mEASIX)	1.325 (1.166–1.506)	<0.001	1.347 (1.172–1.548)	<0.001	1.287 (0.930–1.782)	0.128
Age (per year)	1.040 (1.015–1.065)	0.002	0.997 (0.968–1.027)	0.862	1.038 (0.985–1.093)	0.166
IPSS-R risk group *	1.461 (1.124–1.898)	0.005	1.116 (0.837–1.489)	0.453	1.335 (0.718–2.480)	0.361
HMA exposure	0.423 (0.239–0.750)	0.003	0.627 (0.326–1.206)	0.162	0.442 (0.148–1.321)	0.144
Venetoclax exposure	1.477 (0.811–2.690)	0.203	0.991 (0.505–1.943)	0.978	0.804 (0.098–6.602)	0.839

* IPSS-R risk group was entered into the model as an ordinal variable. HMA and venetoclax were included as binary exposure covariates to partially account for treatment heterogeneity. Landmark analysis was performed at 29 months. HR, hazard ratio; CI, confidence interval; mEASIX, modified Endothelial Activation and Stress Index; IPSS-R, Revised International Prognostic Scoring System; HMA, hypomethylating agent.

**Table 6:** Model performance, validation, and proportional hazards assessment.

Parameter	Primary Model Comparison	Treatment-Adjusted Model Comparison
Likelihood ratio test for addition of log(mEASIX)	χ^2^ = 19.292, *p* = 1.122 × 10^−5^	χ^2^ = 19.505, *p* = 1.003 × 10^−5^
Apparent C-index without log(mEASIX)	0.699	0.725
Apparent C-index with log(mEASIX)	0.748	0.756
Optimism-corrected C-index	0.736	0.739
Bootstrap-corrected calibration slope	0.912	0.869
Global proportional hazards test	*p* = 0.036	*p* = 0.058
Global proportional hazards test after IPSS-R stratification	*p* = 0.31	*p* = 0.70

Primary model comparison: age, IPSS-R versus age, IPSS-R, log(mEASIX). Treatment-adjusted model comparison: age, IPSS-R, HMA exposure and venetoclax exposure versus age, IPSS-R, HMA exposure, venetoclax exposure, log(mEASIX). The C-index was calculated from Somers’ Dxy using the formula C-index = (Dxy + 1)/2. mEASIX, modified Endothelial Activation and Stress Index; IPSS-R, Revised International Prognostic Scoring System.

## Data Availability

The data presented in this study are available on request from the corresponding author.
